# Development of Stearic Acid Nanoemulsion for Therapeutic Delivery of Talazoparib Against Breast Cancer

**DOI:** 10.3390/pharmaceutics18030378

**Published:** 2026-03-19

**Authors:** Jingjing Zhang, Zhongkun Zhang, Xiaohan Xia, Kaixin Feng, Siyu Yao, Yufei Wang, Min Wu

**Affiliations:** 1 Division of Chemical Engineering, School of Chemistry and Chemical Engineering, Southeast University, Nanjing 211189, China; 2 Department of Pharmacology, School of Medicine, Southeast University, Nanjing 211189, China; 3 Department of Medical Oncology, Dana-Farber Cancer Institute, Harvard Medical School, Boston, MA 02115, USA; 4 Key Laboratory of Environmental Medicine Engineering, Ministry of Education, School of Public Health, Southeast University, Nanjing 211189, China

**Keywords:** synthetic BRCAness, DNA damage repair, targeted delivery, long-chain fatty acids, stearic acid, chemosensitization

## Abstract

**Objectives**: Talazoparib (TZL) is a potent PARP inhibitor but suffers from poor aqueous solubility, dissolution-limited absorption, and dose-limiting systemic toxicities, which together restrict its antitumor efficacy in some breast cancer settings. This study aimed to develop a stearic acid-based nanoemulsion (SANE) to improve the delivery of TZL and enhance its antitumor activity and preliminarily explore its impact on DNA damage response-related pathways. **Methods**: SANE-TZL was prepared using a high-pressure homogenization method, and its physicochemical properties were characterized. MCF-7 and MDA-MB-231 breast cancer cells were used to evaluate cellular uptake, cytotoxicity, and changes in key DNA damage response markers. In vivo therapeutic efficacy and safety were assessed in an MDA-MB-231 xenograft mouse model. **Results**: SANE-TZL exhibited a uniform particle size of approximately 118 nm with excellent stability. In MCF-7 cells, SANE-TZL significantly enhanced drug internalization, resulting in an 8.4-fold reduction in IC_50_ compared to free TZL. Consistently, in MDA-MB-231 cells, SANE-TZL also showed markedly increased antiproliferative activity. At the molecular level, SANE-TZL modulated the expression of several DNA damage response-related genes, including *BRCA1*, *RAD51*, and *SLFN11*, in a manner consistent with impaired DNA repair capacity. In vivo, high-dose SANE-TZL achieved a tumor growth inhibition (TGI) rate of 58.55%, which was higher than that of the free TZL group (41.86%) and the blank eSANE group (17.59%). No evident hematological or organ toxicities were observed in the SANE-TZL-treated groups. **Conclusions**: SANE-TZL markedly improves the delivery efficiency and antitumor activity of TZL in breast cancer models while maintaining a favorable safety profile. By combining a functional stearic acid carrier with TZL, this nanoemulsion formulation represents a safe and potent strategy to enhance PARP inhibitor-based chemotherapy in breast cancer, and it may provide a basis for further mechanistic studies on DNA damage response modulation.

## 1. Introduction

Breast cancer is a highly heterogeneous malignancy and remains a major threat to women’s health worldwide. Clinically, treatment strategies are largely guided by the expression status of three key receptors: estrogen receptor (ER), progesterone receptor (PR), and human epidermal growth factor receptor 2 (HER2) [1]. At the molecular level, the breast cancer susceptibility genes BRCA1 and BRCA2 (BRCA1/2) are critical for maintaining genomic stability by repairing DNA double-strand breaks through the homologous recombination (HR) pathway [2,3]. Loss or functional inactivation of BRCA1/2 forces cells to rely on error-prone non-homologous end joining (NHEJ), leading to genomic instability and increased vulnerability to DNA-damaging agents [4,5]. Triple-negative breast cancer (TNBC), which frequently correlates with BRCA1 mutations or a “BRCAness” phenotype, remains particularly challenging due to the lack of targetable receptors; chemotherapy is still the mainstay of treatment, yet limited drug accumulation at the tumor site often results in resistance and poor outcomes [6]. Poly(ADP-ribose) polymerase (PARP) inhibitors, which exploit synthetic lethality in BRCA-deficient cells, have therefore emerged as a rational strategy for treating BRCA-mutated breast cancers [7,8].

Talazoparib (TZL) is a next-generation PARP inhibitor with approximately 100-fold greater PARP–DNA trapping potency than first-generation agents such as Olaparib [9]. In germline BRCA-mutated breast cancer, TZL has demonstrated meaningful clinical benefit; however, in broader breast cancer populations, including some BRCA-proficient tumors, its therapeutic effect can be limited by pharmacokinetic and toxicity constraints rather than target engagement alone [10]. TZL is a Biopharmaceutics Classification System (BCS) Class II drug, characterized by extremely low aqueous solubility and dissolution-limited absorption [11,12]. Clinically, TZL treatment is frequently accompanied by dose-limiting hematological toxicities and other adverse effects, while preclinical studies report an oral bioavailability of only about 56% in rats [13]. These factors restrict dose escalation and long-term administration, limiting the opportunity to extend TZL therapy beyond the current BRCA-mutant population. Thus, there is a pressing need for delivery strategies that can improve TZL solubility and bioavailability, enhance its tumor-selective accumulation, and reduce systemic toxicity.

To address the delivery challenges of TZL, several nano-platforms have been explored. For instance, Zhang et al. developed PEGylated nano-liposomes [14] to enhance the therapeutic index; however, these lipid vesicles exhibit a relatively low drug-loading capacity and inherent structural instability, which can lead to premature drug leakage and fail to shield patients from TZL-induced hematological toxicities adequately. Similarly, while Baldwin et al. utilized poly(lactic-co-glycolic acid) (PLGA) nanoparticles and solid lipid nanoparticles (SLNs) [15] to increase the maximum tolerated dose of TZL, these systems often face challenges in large-scale production (scalability) and physical stability, such as particle aggregation over time. Notably, that study also revealed a pronounced “burst release” effect, where approximately 30–50% of the encapsulated TZL was rapidly released within the first few hours. This rapid initial release into the systemic circulation potentially leads to off-target effects and limits the amount of drug that actually reaches the tumor site.

In contrast, nanoemulsions (NEs) offer a superior solubilizing capacity for BCS Class II drugs and, crucially, can be manufactured using high-pressure homogenization, a process highly compatible with industrial-scale production. Among various oil phases, delivery systems based on long-chain fatty acids (LCFAs) have been reported to possess intrinsic antitumor activity arising from the carrier itself [16,17,18]. In particular, the selection of stearic acid (SA), a saturated LCFA, provides a unique dual functionality. Unlike liquid-state unsaturated oils (e.g., oleic acid) that are prone to Ostwald ripening or lipid peroxidation, SA forms a more rigid and hydrophobic matrix at physiological temperatures, effectively encapsulating TZL to minimize burst release and ensure superior physicochemical stability. Beyond its role as a structural scaffold, SA has been shown to selectively induce apoptosis and growth inhibition in breast cancer cells through the induction of endoplasmic reticulum (ER) stress [19,20], thereby offering synergistic potential to overcome the limitations of conventional inert carriers. Nevertheless, the design of LCFA-based nanoemulsions for systemic cancer therapy still faces challenges related to long-term physicochemical stability, controlled drug release, and scalable, reproducible preparation processes [21].

Building upon the therapeutic potential and delivery advantages of LCFAs, we developed a series of long-chain fatty acid nanoemulsions (LCFANEs) as delivery platforms for TZL in breast cancer therapy. By systematically comparing nanoemulsions based on different fatty acid cores, we identified a stearic acid-based nanoemulsion (SANE) exhibiting favorable stability, pH-modulated release, and efficient cellular uptake. We then evaluated the sensitizing effect of SANE-loaded talazoparib (SANE-TZL) to enhance the antiproliferative and pro-apoptotic effects of TZL in breast cancer cell lines and investigated associated changes in key DNA damage response-related genes. Furthermore, we validated the in vivo antitumor efficacy and safety of SANE-TZL in tumor-bearing mouse models. Together, these findings suggest that SANE-TZL not only improves the delivery and antitumor efficacy of TZL but also provides a versatile platform for further exploration of PARP inhibitor-based combination or sensitization strategies in breast cancer.

## 2. Materials and Methods

### 2.1. Materials

TZL, oleic acid (OA), linoleic acid (LA), SA, Tween-80, dimethylsulfoxide (DMSO), and anhydrous ethanol were purchased from Myriad Technologies Ltd. (Shanghai, China). Di-stearoyl phosphatidylcholine (DSPC) and deionized water were purchased from McLean Biochemical Technology Co., Ltd. (Shanghai, China). Hydrochloric acid (HCl), chloroform (CHCl_3_), and isopropanol (IPA, C_3_H_8_O) were purchased from Sinopharm Chemical Reagent Co., Ltd. (Shanghai, China). DMEM, serum, CCK-8 reagent, Trizol reagent, DEPC water, and phosphate-buffered solution (PBS, 1×, pH 7.4) were purchased from KeyGEN Biotechnology Co. (Nanjing, China). Reverse transcription reagent HiScript III was purchased from Nanjing Novozymes Biotech Co., Ltd. (Vazyme R333-C1, Nanjing, China), and SYBR green master mix was purchased from BioEasy BSB113S1 (Hangzhou, China).

### 2.2. Synthesis of Different LCFANE-TZL

LCFANE-TZL was synthesized using DSPC, Tween-80, a type of LCFAs (OA, LA, and SA), and TZL. The lipophilic phase was prepared by dissolving DSPC, Tween-80, and LCFA in anhydrous ethanol at a mass percentage of 55:20:25; The detailed compositions of these formulations are summarized in Table 1. TZL was mixed in the lipophilic phase with a drug–NE weight ratio of 1:10, and the mixed solution was rapidly injected into PBS using the solvent injection method, followed by probe sonication to generate LCFANE-TZL. Empty LCFANE (eLCFANE) was prepared according to the same method described above, but without the addition of TZL. The above eLCFANE and LCFANE-TZL were prepared at a batch volume of 1 mL for each formulation.

### 2.3. Characterization Methods

The morphology and size of the nanoemulsions were observed using transmission electron microscopy (TEM) after negative staining with 1.0% (*w*/*v*) phosphotungstic acid. The hydrodynamic diameter, polydispersity index (PDI), and zeta potential of the nanoemulsions were measured using a Brookhaven 90 Plus analyzer equipped with ZetaPALS (Brookhaven Instruments Corp., Holtsville, NY, USA).

The encapsulation efficiency (EE%) of TZL in LCFANE-TZL was determined by the dialysis method. Briefly, 1 mL of freshly prepared LCFANE-TZL was placed in a dialysis bag (molecular weight cut-off 3500 Da) and dialyzed against 10 mL of pre-equilibrated phosphate-buffered saline (PBS, pH 7.4) at 37 °C with gentle shaking (100 rpm) for 4 h. The concentration of free TZL in the external dialysis medium was quantified by measuring absorbance at 311 nm using a Pono-550 spectrophotometer (Porabio Instrument (Hangzhou) Co., Ltd.; Hangzhou, China), based on a pre-established standard curve (linear range 0.1–5.0 mg/mL, R^2^ ≥ 0.99).

EE% was calculated according to the following equation:

(1) $$EE,\%\;=\frac{m_{1}-(C_{free}\;\times \;V_{external})}{m_{1}}\;\times \;100\%$$

where *m*_1_ is the total mass of TZL in the 1 mL nanoemulsion before dialysis, *C_free_* is the concentration of free TZL in the external medium, and *V_external_* is the volume of the external dialysis medium.

Drug loading (DL%) was defined as the mass percentage of encapsulated TZL relative to the total mass of the LCFANE-TZL nanoemulsion and was calculated based on the theoretical drug feeding ratio of 9% (*w*/*w*). All measurements were performed in triplicate, and results are expressed as mean ± standard deviation (SD). The long-term stability of LCFANE-TZL was evaluated by monitoring changes in particle size at predetermined time points for up to 30 days.

### 2.4. In Vitro Drug Release Studies

The in vitro release profile of TZL from LCFANE-TZL was evaluated using the dialysis bag method. Briefly, 1 mL of LCFANE-TZL was accurately transferred into a dialysis bag with a molecular weight cut-off (MWCO) of 3500 Da. The sealed dialysis bag was immersed in 10 mL of PBS release medium (pH 5.0, 6.5, or 7.4, adjusted with 1 M HCl) and placed in a constant temperature shaker at a stirring speed of 100 rpm. Two incubation temperatures were set: 4 °C (simulating storage conditions) and 37 °C (simulating physiological conditions).

At predetermined time intervals (0.25, 0.75, 2.0, 4.0, 6.0, 12.0, and 24.0 h), 1 mL aliquot of the external release medium was withdrawn for analysis. To maintain sink conditions, an equal volume (1 mL) of fresh pre-warmed (37 °C) or pre-cooled (4 °C) PBS (corresponding to the incubation temperature) was immediately added back to the release system after each sampling. The concentration of TZL in the collected samples was determined using a Nanodrop spectrophotometer (Thermo Fisher Scientific, Waltham, MA, USA), following the method described in Section 2.3 (UV absorbance at 311 nm). The cumulative release percentage of TZL was calculated according to the following equation:

(2) $$TZL\;released,\;\%\;=\;\frac{C_{i}\;\times \;V_{buf}\;+\;\sum_{i=1}^{n-1}\left(C_{i-1}\;\times \;V_{s}\right)}{M}\;\times \;100\%$$

where *V_buf_* is the volume of dialysis buffer, *C_i_* is the concentration of TZL in the release medium at each sampling time point, *V_s_* is the sampling volume (1 mL), and *M* is the total amount of TZL initially loaded in the nanoemulsion. All experiments were performed in triplicate, and results are expressed as mean ± standard deviation (SD).

### 2.5. Cell Culture

MCF-7 and MDA-MB-231 human breast cancer cells were cultured in DMEM containing 10% FBS supplemented with 1% penicillin–streptomycin in a humidified incubator at 37 °C with 5% CO_2_. Cells were maintained in the logarithmic growth phase and used for experiments when they reached approximately 70–80% confluence.

### 2.6. Cellular Uptake Assay

MCF-7 and MDA-MB-231 cells were seeded in 96-well plates at a density of 5 × 10^3^ cells per well and incubated overnight for attachment. The cellular uptake of LCFANE-TZL was evaluated using two complementary approaches based on both a fluorescent tracer and the intrinsic fluorescence of TZL [22].

For qualitative observation of uptake behavior, the lipophilic fluorescent dye DiO (50 nM) was added to LCFANE-TZL and free TZL formulations at a volume ratio of 1% (*v*/*v*). Cells were treated with DiO-labeled formulations (equivalent to 30 μM TZL) and incubated in the dark for 48 h.

For absolute quantitative determination of intracellular TZL concentration, a matrix-matched linear fluorescence standard curve was established in blank cell lysates. Briefly, blank lysates were prepared from untreated MCF-7 and MDA-MB-23 cells. TZL calibration standards ranging from 1 to 200 ng/mL were prepared in the blank lysates. Fluorescence intensity was measured using a fluorescence microplate reader (Shanghai Flash Spectrum Biotechnology Co., Ltd.; Shanghai, China) at λex = 360 nm and λem = 465 nm, under the same instrument parameters as those used in the cellular uptake experiment. The standard curve was validated with three independent batches (R^2^ ≥ 0.99).

For quantitative uptake analysis, cells were treated with free TZL or LCFANE-TZL (30 μM TZL equivalent) for 24, 48, and 72 h. After incubation, the medium was removed, and the cells were washed three times with ice-cold PBS to eliminate the extracellular drug. Cells were then lysed and centrifuged, and the fluorescence intensity of the supernatant was measured. The intracellular TZL concentration was calculated by interpolation from the validated standard curve. The number of cells per well was counted to normalize the data. Results are expressed as ng TZL per 10^6^ cells. All experiments were performed in triplicate.

### 2.7. Cytotoxicity Assay

The cytotoxicity of free TZL and LCFANE-TZL toward MCF-7 and MDA-MB-231 cells was evaluated using a Cell Counting Kit-8 (CCK-8) assay, with untreated cells serving as the control. Cells were seeded in 96-well plates at a density of 5 × 10^3^ cells per well and allowed to attach for 24 h. The culture medium was then replaced with fresh medium containing free TZL or LCFANE-TZL at concentrations ranging from 0 to 200 μM. After incubation for 48 or 72 h, 100 μL of DMEM containing 10% (*v*/*v*) CCK-8 reagent (KeyGEN BioTECH Co., Nanjing, China) was added to each well and incubated for a further 2 h. Absorbance was measured at 450 nm using a microplate reader (Multiskan GO 1510, Thermo Fisher Scientific, Shanghai, China). Cell viability was calculated according to the following equation:

(3) $$\mathrm{Cell}\;\mathrm{Viability},\;\%=\frac{{\mathrm{OD}}_{\mathrm{test}}-{\mathrm{OD}}_{\mathrm{bare}}}{{\mathrm{OD}}_{\mathrm{control}}-{\mathrm{OD}}_{\mathrm{bare}}}\;\times \;100\%$$

where *OD_bare_* is the absorbance of wells containing CCK-8 reagent and medium without cells (and the corresponding amount of drug vehicle), *OD_control_* is the absorbance of untreated control cells, and *OD_test_* is the absorbance of cells treated with free TZL or LCFANE-TZL. The viability of untreated cells was normalized to 100%, and half-maximal inhibitory concentration (IC_50_) values for each formulation and cell line were obtained by fitting the cell viability–concentration curves using nonlinear regression.

### 2.8. Flow Cytometry

MCF-7 cells were seeded in 6-well plates at a density of 5 × 10^5^ cells per well and incubated overnight in complete growth medium at 37 °C with 5% CO_2_. Cells were then treated with free TZL (150 μM) or LCFANE-TZL (30 μM TZL equivalent) for 24 h. After treatment, cells were harvested, washed twice with cold PBS, and stained with Annexin V–FITC/propidium iodide (PI) using a commercial apoptosis detection kit (KeyGEN BioTECH Co., Nanjing, China) according to the manufacturer’s instructions. Apoptosis was analyzed on a BD Accuri™ C6 Plus flow cytometer (BD Biosciences, San Jose, CA, USA), and the percentages of viable, early apoptotic, late apoptotic, and necrotic cells were quantified.

### 2.9. RT-qPCR Analysis

To evaluate changes in gene expression in breast cancer cells after treatment with LCFANE-TZL, reverse transcription–quantitative polymerase chain reaction (RT-qPCR) was performed using RNA extracted from MCF-7 and MDA-MB-231 cells. Cells were treated with the indicated formulations under the same conditions as in the cytotoxicity assays and then harvested for RNA isolation. Total RNA was extracted using Trizol reagent (KeyGEN BioTECH Co., China) according to the manufacturer’s protocol, and RNA concentration and purity were determined with a Porabio Pono-500 spectrophotometer. Complementary DNA (cDNA) was synthesized from 1 μg of total RNA using HiScript III reverse transcriptase (Vazyme, R333-C1, Nanjing, China). Gene-specific primers (sequences listed in Table S1) were synthesized by KeyGEN BioTECH Co. RT-qPCR was conducted on a real-time fluorescence quantitative PCR system (Bioer FQD-96A, Hangzhou, China) using SYBR Green SuperMix (BioEasy BSB113S1, Hangzhou, China). Relative mRNA expression levels were calculated using the 2^−ΔΔCt^ method, with GAPDH serving as the internal reference gene.

### 2.10. In Vivo Antitumor Studies

The in vivo antitumor efficacy of SANE-TZL was evaluated in a human triple-negative breast cancer xenograft model established with MDA-MB-231 cells. MDA-MB-231 is a highly invasive, BRCA-proficient triple-negative breast cancer (TNBC) cell line that exhibits clinical features of aggressive disease and is commonly used to model refractory breast cancer in vivo, making it suitable for assessing the therapeutic potential of PARP inhibitor-based formulations in a BRCA-proficient context [23,24]. Female BALB/c nude mice were subcutaneously inoculated in the right axilla with 0.1 mL of an MDA-MB-231 cell suspension (5 × 10^6^ cells per mouse) to establish tumors (all cells and animals used in this study were obtained from KeyGEN BioTECH Co., Nanjing, China). All animal experiments were conducted in compliance with institutional and national guidelines for the care and use of laboratory animals. The study protocol was reviewed and approved by the Ethics Committee of Nanjing Advanced Academy of Life and Health (Animal Welfare Assurance Number: NAALH-W-2504030). All procedures involving BALB/c nude mice were performed under the Experimental Animal Production License (SCXK 2022-0006, Certificate No. A202505160430) and the Experimental Animal Use License (SYXK 2023-0001).

Treatment was initiated when tumor volumes reached approximately 80–100 mm^3^. Tumor-bearing mice were randomly assigned to six groups (*n* = 6 per group): (1) saline (control, i.v.); (2) Free-TZL (i.v., 0.33 mg/kg); (3) eSANE (i.v.); (4) SANE-TZL-low (i.v., 0.11 mg/kg TZL equivalent); (5) SANE-TZL-middle (i.v., 0.33 mg/kg TZL equivalent); and (6) SANE-TZL-high (i.v., 1.00 mg/kg TZL equivalent) [25,26,27]. Free TZL and SANE-TZL formulations were diluted to the desired working concentrations with PBS immediately before administration. Treatments were given via tail vein injection every other day at a dose volume of 200 μL per mouse. The dosing schedule lasted for 18 days, after which mice were euthanized for tissue collection.

Body weight and tumor size were recorded before each injection. Tumor length and width were measured with a digital caliper, and tumor volume was calculated according to the following formula:

(4) $$\mathrm{Tumor}\;\mathrm{Volume}\;=\;\frac{\mathrm{Length}\;\times \;{\mathrm{Width}}^{2}}{2}$$

Relative tumor volume (RTV) was defined as follows:

(5) $$\mathrm{RTV}\;=\;\frac{\mathrm{tumor}\;\mathrm{volume}\;\mathrm{at}\;\mathrm{measurement}}{\mathrm{initial}\;\mathrm{tumor}\;\mathrm{volume}}$$

Terminal tumor growth inhibition (TGI, %) was calculated using the following equation:

(6) $$\mathrm{TGI},\;\%\;=\;\frac{{\mathrm{RTV}}_{\mathrm{comtrol}\;}-{\;\mathrm{RTV}}_{\mathrm{test}}}{{\mathrm{RTV}}_{\mathrm{control}}}\;\times \;100\%$$

where *RTV_control_* and *RTV_test_* are the mean relative tumor volumes of the control and treatment groups, respectively.

### 2.11. Hematoxylin and Eosin (H&E) Staining and Biochemistry Parameters

At the end of the in vivo study, mice were anesthetized, and blood was collected via orbital sinus puncture to prevent coagulation. Immediately thereafter, humane euthanasia was performed by cervical dislocation. After death was confirmed, a sterile necropsy was conducted to excise tumors and major organs (liver, lung, heart, kidney, and spleen), which were processed according to the experimental requirements.

Tumor and organ tissues were fixed in 10% neutral buffered formalin, embedded in paraffin, sectioned, and stained with hematoxylin and eosin (H&E) for histopathological evaluation. Whole blood samples were allowed to clot at room temperature for 2 h and then centrifuged at 3000 rpm for 15 min at 2–8 °C. The resulting serum was collected for immediate biochemical analysis of hepatic and cardiac function markers.

### 2.12. Statistical and Data Analysis

All studies were done in triplicate unless otherwise specified. Data are presented as means ± standard deviations unless otherwise indicated. Statistical analysis was conducted using GraphPad Prism 10 (version 10.5.0, GraphPad Software Inc., Boston, MA, USA). One-way ANOVA was used to determine variances in means between two or more treatment groups. Tukey’s Honestly Significant Difference (HSD) test was further used as a post-hoc analysis to determine statistically significant differences after one-way ANOVA. Student’s *t*-test was performed in case of comparing the statistical differences between two groups of interest only. A *p*-value of 0.05 was selected as the cut-off for statistical significance. In addition to statistical software, FlowJo (version 10.8.1, Becton, Dickinson and Company, Ashland, OR, USA) was utilized for flow cytometric data analysis. Histological and HE-stained images were processed and analyzed using OLYVIA software (version 3.1.1, Evident Corporation, Tokyo, Japan). All scientific illustrations and figures were prepared using Adobe Illustrator 2026 (version 30.2.1, Adobe Inc., San Jose, CA, USA).

## 3. Results

### 3.1. Characterization of LCFANE-TZL

To ensure accurate characterization and optimal drug loading, the analytical methods and formulation parameters were first established. As shown in Figure S1a, a linear standard curve for TZL was obtained, providing the basis for quantitative analysis. The dialysis time for encapsulation efficiency (EE%) determination was pre-optimized at 4 h, ensuring the complete removal of unencapsulated drugs (Figure S1b). Based on these methods, the effects of various drug-to-lipid ratios (DLRs) on particle size and EE% were systematically screened (Figure S1c,d). Considering the particle size and stability of the nanoparticles, as well as the clinical applicability of administration volume and the economic efficiency of the formulation, the optimal lipid–surfactant–fatty acid ratio (LSFR) and drug lipid ratio (DLR) were determined to be 55:20:25 and 1:10, respectively.

**Figure 1:**
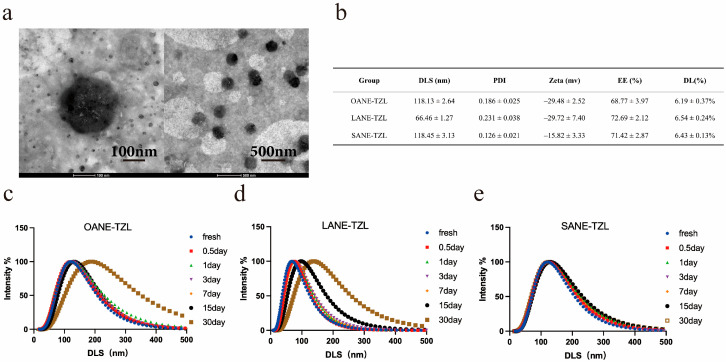
Characterization and stability of LCFANE-TZL: (**a**) TEM micrographs revealing the spherical morphology of SANE-TZL. (**b**) Physicochemical properties (DLS, PDI, zeta potential, EE%, and DL%) of freshly prepared OANE-TZL, LANE-TZL, and SANE-TZL. (**c**–**e**) Time-dependent changes in particle size distribution for (**c**) OANE-TZL, (**d**) LANE-TZL, and (**e**) SANE-TZL during storage at 4 °C for 30 days.

The physicochemical properties of the optimized OANE-TZL, LANE-TZL, and SANE-TZL were further characterized. As shown in Figure 1b, all freshly prepared nanoemulsions exhibited relatively small and uniform particle sizes. Specifically, the average hydrodynamic diameters of OANE-TZL and SANE-TZL were 118.13 ± 2.64 nm and 118.45 ± 3.13 nm, respectively, while LANE-TZL displayed the smallest initial size of 66.46 ± 1.27 nm. The PDI for all formulations ranged from 0.126 to 0.231, indicating a narrow size distribution and excellent dispersibility. The zeta potentials were found to be negative, ranging from −15.82 mV to −29.72 mV, which provides essential electrostatic repulsion for maintaining system stability. The zeta potentials were found to be negative, with peak values recorded at −29.48 ± 2.52 mV (OANE-TZL), −29.72 ± 7.40 mV (LANE-TZL), and −15.82 ± 3.33 mV (SANE-TZL), providing essential electrostatic repulsion for maintaining system stability. Furthermore, all nanoemulsions achieved high encapsulation efficiency (EE%) and drug loading (DL%), with SANE-TZL reaching an EE% of 71.42 ± 2.87% and a DL% of 6.43 ± 0.13%, consistent with the theoretical DLR. TEM imaging (Figure 1a) further confirmed that these nanoemulsions maintained a well-defined spherical morphology with size distributions highly consistent with the DLS measurements.

To assess the long-term physical stability, the particle size variations were monitored over a 30-day storage period at 4 °C (Figure 1c–e). The results revealed significant differences in stability among the formulations. The particle sizes of OANE-TZL and LANE-TZL increased substantially from their initial values to 184.19 nm and 135.24 nm, respectively, accompanied by a noticeable broadening of the distribution peaks. In contrast, SANE-TZL maintained superior stability, with its particle size only slightly increasing to 128.64 nm after 30 days. TEM imaging (Figure 1a) further confirmed that SANE-TZL maintained a well-defined spherical morphology with a size distribution consistent with the DLS measurements. These findings suggest that the SANE provides a more robust matrix for drug encapsulation and long-term storage compared to its unsaturated counterparts.

### 3.2. In Vitro Drug Release Study of LCFANE-TZL

**Figure 2:**
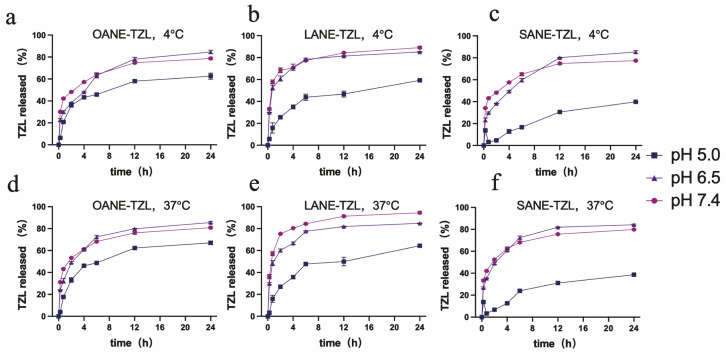
In vitro drug release profiles of LCFANE-TZL under various conditions: (**a**–**c**) Cumulative release of TZL from (**a**) OANE-TZL, (**b**) LANE-TZL, and (**c**) SANE-TZL at 4 °C across different pH values (5.0, 6.5, and 7.4) over 24 h. (**d**–**f**) Cumulative release of TZL from (**d**) OANE-TZL, (**e**) LANE-TZL, and (**f**) SANE-TZL at 37 °C across different pH values (5.0, 6.5, and 7.4) over 24 h. Data are presented as mean ± SD (*n* = 3).

The drug release behavior of LCFANE-TZL was systematically evaluated under varying pH (7.4, 6.5, and 5.0) and temperature (4 °C and 37 °C) conditions to simulate the complex physiological journey of the nanocarrier. As illustrated in Figure 2, the cumulative release profiles of all formulations at 4 °C and 37 °C were nearly identical (*p* > 0.05), validating the robust thermal stability and structural integrity of the nanoemulsion platforms.

The SANE-TZL formulation exhibited a distinct pH-modulated sustained-release characteristic. At pH 7.4 (simulating physiological blood) and pH 6.5 (simulating the acidic tumor interstitium), SANE-TZL achieved high release efficiency, with 24 h cumulative release rates reaching 79.85% and 84.19% at 37 °C, respectively. Notably, when the pH was further decreased to 5.0 (mimicking the endo-lysosomal environment), the release rate of SANE-TZL significantly declined to 38.72%. This decelerated release at lower pH distinguished SANE-TZL from the other two groups, OANE-TZL (67.07%) and LANE-TZL (64.40%), highlighting the unique responsiveness of the stearic acid-based system.

### 3.3. Breast Cancer Cell Killing Efficacy of LCFANE-TZL

To further elucidate the impact of different long-chain fatty acid (LCFA) carriers on TZL efficacy, we investigated the cellular uptake behavior in MCF-7 cells using complementary qualitative and quantitative approaches. Qualitative tracing via DiO-labeled formulations (50 nM, 1% *v*/*v*) revealed that while Free-TZL showed negligible fluorescence after 48 h of incubation, all LCFANE-TZL groups exhibited distinct fluorescence accumulation. Notably, the SANE-TZL group displayed the most intense intracellular signal, suggesting superior internalization (Figure S2a).

For rigorous quantification, a matrix-matched linear fluorescence standard curve (R^2^ = 0.9982) was established to ensure the accuracy of intracellular TZL determination (Figure S2b). The quantitative results confirmed a clear time-dependent uptake profile for all formulations (Figure S2c). Throughout the observation period, SANE-TZL consistently maintained the highest intracellular drug accumulation. This advantage became increasingly prominent over time; at 72 h, the intracellular concentration of TZL in the SANE-TZL group reached its peak at 597.72 ± 14.90 ng/10^6^ cells, which was approximately 4.9-fold higher than that of the Free-TZL group (121.92 ± 3.82 ng/10^6^ cells), 1.4-fold higher than the LANE-TZL group (419.43 ± 20.36 ng/10^6^ cells), and 1.3-fold higher than the OANE-TZL group (466.10 ± 9.17 ng/10^6^ cells) (Figure S2c). These findings demonstrate that the stearic acid-based nanoemulsion significantly enhances the sustained delivery and superior retention of TZL within breast cancer cells.

**Figure 3:**
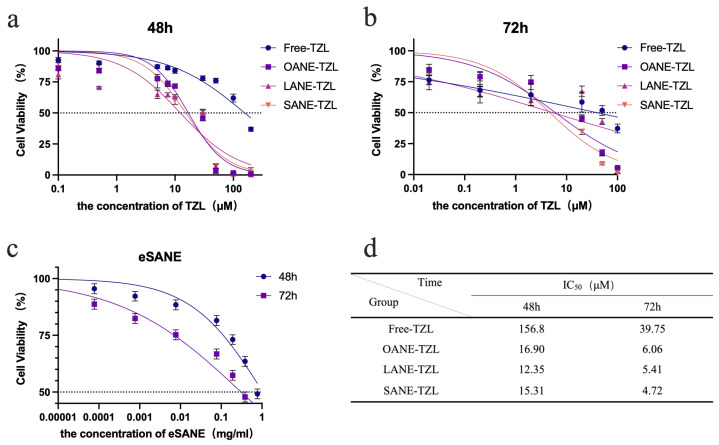
Cytotoxicity of Free-TZL and LCFANE-TZL in MCF-7 cells: (**a**,**b**) Cell viability of MCF-7 cells after treatment with Free-TZL and various LCFANE-TZL formulations for (**a**) 48 h and (**b**) 72 h, determined by CCK-8 assay. (**c**) Cell viability after treatment with eSANE for 48 h and 72 h. (**d**) IC_50_ values of Free-TZL and LCFANE-TZL at 48 h and 72 h. The horizontal dashed line indicates 50% cell viability used for the determination of IC_50_ values. Data are presented as mean ± SD (*n* = 3).

The growth inhibitory effects of LCFANE-TZL on MCF-7 cells were evaluated using the CCK-8 assay (Figure 3). All drug-loaded formulations exhibited concentration-dependent cytotoxicity. After 72 h incubation, Free-TZL showed relatively low antitumor activity with an IC_50_ of 39.75 μM. Nanoemulsion encapsulation significantly enhanced the killing efficiency; the IC_50_ values of OANE-TZL and LANE-TZL decreased to 6.06 μM and 5.41 μM, respectively. Most notably, SANE-TZL demonstrated the strongest inhibitory effect with an IC_50_ as low as 4.72 μM, representing an 8.4-fold increase in potency compared to Free-TZL. The blank eSANE carrier showed no significant cytotoxicity, confirming its biological safety. Parallel experiments in MDA-MB-231 TNBC cells demonstrated consistent sensitization (Figure S3), with SANE-TZL achieving an IC_50_ of 2.40 μM at 72 h.

### 3.4. Mechanisms Underlying the Cancer Cell Killing Effects

**Figure 4:**
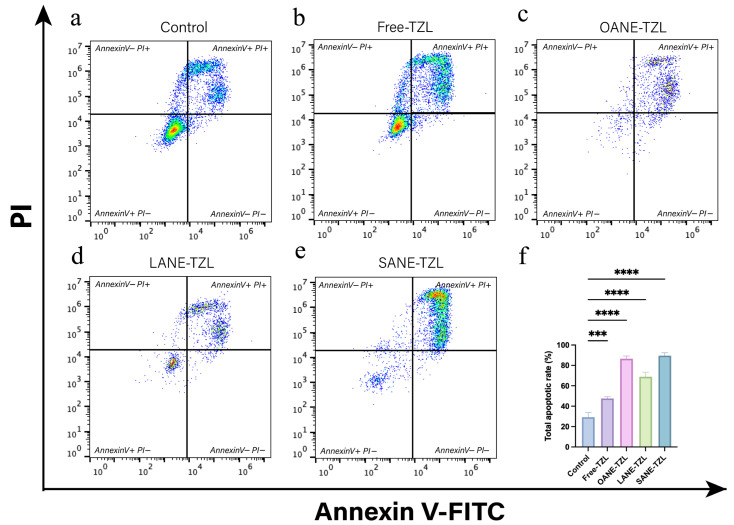
Flow cytometry analysis of apoptosis in MCF-7 cells: (**a**–**e**) Representative Annexin V-FITC/PI flow cytometry plots of MCF-7 cells following treatment with (**a**) control, (**b**) Free-TZL, (**c**) OANE-TZL, (**d**) LANE-TZL, and (**e**) SANE-TZL for 72 h. (**f**) Statistical analysis of the total apoptotic rates (sum of early and late apoptosis) for each group. The color density in the plot represents the cell population density, ranging from blue (low density) to red (high density). Data are presented as mean ± SD (*n* = 3). Significant differences are indicated as *** *p* < 0.001 and **** *p* < 0.0001.

Mechanistic insights into the pro-apoptotic effects were obtained via Annexin V-FITC/PI flow cytometry (Figure 4). Free-TZL induced a total apoptosis rate of 47.65 ± 1.61%, while the nanoemulsion groups showed markedly stronger effects, with OANE-TZL, LANE-TZL, and SANE-TZL reaching 86.51 ± 2.70%, 68.89 ± 4.40%, and 89.64 ± 2.73%, respectively. Statistical analysis (Table S2) revealed that all LCFANE-TZL formulations significantly increased the apoptotic population compared to both the Control and Free-TZL groups (*p* < 0.001). Notably, SANE-TZL demonstrated the highest apoptotic enhancement, representing a 206.04% increase over the control and an 88.12% increase compared to the Free-TZL group. Although OANE-TZL showed high transient apoptosis, its limited physical stability (Section 3.1) makes SANE-TZL a more promising candidate for clinical translation.

To systematically evaluate the molecular mechanisms underlying the enhanced anticancer efficacy of SANE-TZL, we selected a panel of eight representative genes, focusing on three critical dimensions: (i) DNA damage response (DDR) and repair (*BRCA1*, *RAD51*, *FANCI*, *PARP1*, *SLFN11*) to assess the induction of “synthetic BRCAness”; (ii) apoptosis and cell cycle control (*CASP3*, *CCND1*) to verify the activation of programmed cell death; and (iii) cell adhesion (*CDH1*) to monitor the impact on cellular aggressiveness.

**Figure 5:**
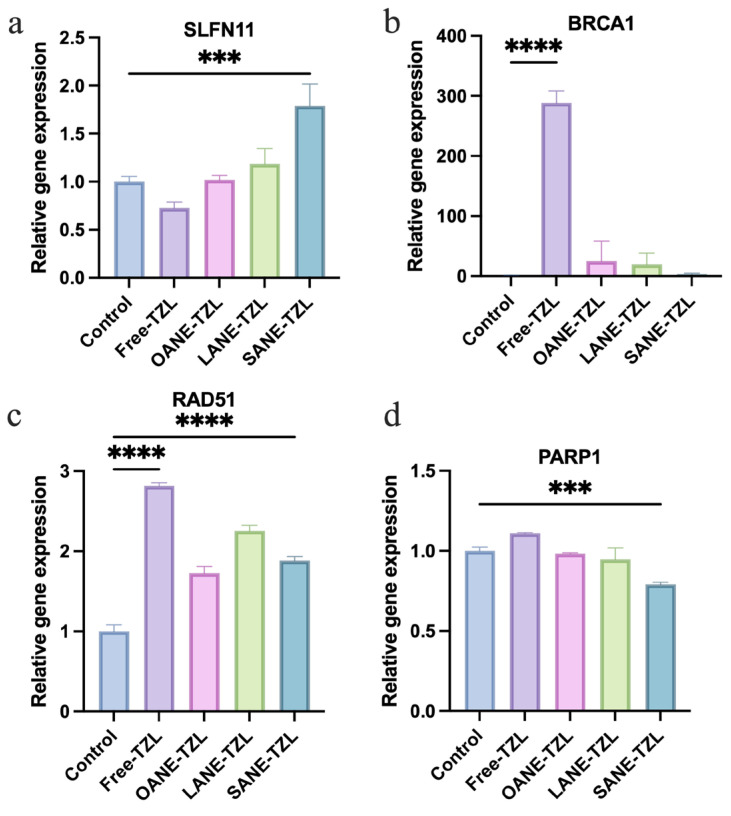
RT-qPCR analysis of gene expression in MCF-7 cells: (**a**–**d**) Relative mRNA expression levels of (**a**) *SLFN11*, (**b**) *BRCA1*, (**c**) *RAD51*, and (**d**) *PARP1* in MCF-7 cells treated with Free-TZL and various LCFANE-TZL formulations. Data are presented as mean ± SD (*n* = 3). Significant differences are indicated as *** *p* < 0.001 and **** *p* < 0.0001.

Molecular analysis (Figure 5) confirmed that SANE-TZL significantly upregulated the DNA damage sensitivity marker *SLFN11* (*p* < 0.001). Regarding DNA repair, while Free-TZL triggered a compensatory surge in *BRCA1* and *RAD51*, SANE-TZL effectively suppressed this self-rescue pathway, successfully inducing a state of “synthetic BRCAness”. Furthermore, the significant elevation of PARP1 mRNA in the SANE-TZL group suggested a robust cellular response to extensive DNA damage, further supporting the potentiation of PARP inhibition-induced genomic instability. Other genes involved in cell cycle regulation, DDR, and cell adhesion (including *CCND1*, *FANCI*, *CASP3*, and *CDH1*) followed consistent trends and are detailed in Figure S4.

### 3.5. In Vivo Efficacy of LCFANE-TZL

**Figure 6:**
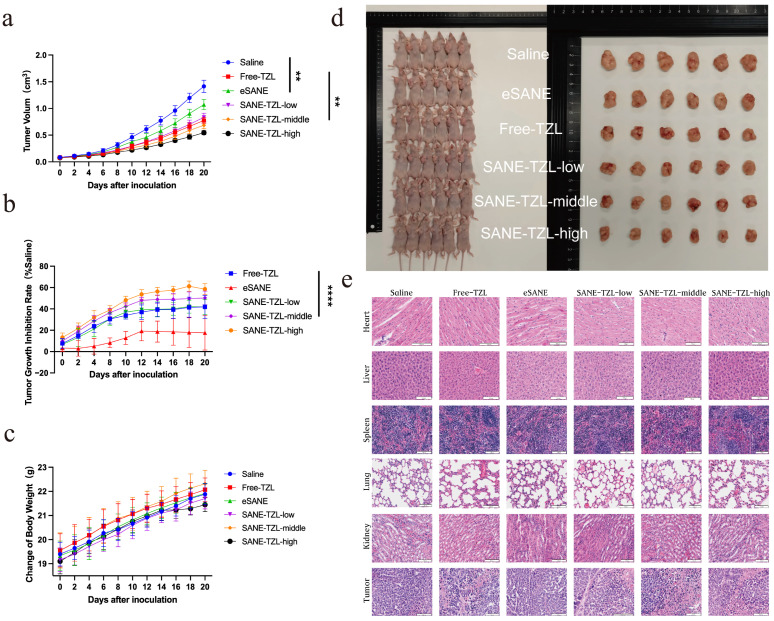
In vivo antitumor activity and biosafety evaluation: (**a**,**b**) Tumor growth kinetics represented by (**a**) tumor volume changes and (**b**) TGI in MDA-MB-231 tumor-bearing mice treated with saline, Free-TZL, eSANE, and SANE-TZL (low, middle, and high doses). (**c**) Body weight changes of mice during the treatment period. (**d**) Digital photographs of mice and excised tumors from each group at the end of the study. (**e**) Representative H&E-stained images of major organs (heart, liver, spleen, lung, and kidney) and tumor tissues. Data are presented as mean ± SD (*n* = 6). Significant differences are indicated as ** *p* < 0.01 and **** *p* < 0.0001. Scale bar = 100 μm.

The therapeutic potential of SANE-TZL was rigorously evaluated in a subcutaneous MDA-MB-231 xenograft tumor-bearing nude mouse model (Figure 6). The results demonstrated that SANE-TZL inhibited tumor growth in a pronounced dose-dependent manner. By the end of the 20-day treatment period, the Free-TZL group (0.33 mg/kg) showed a TGI of 41.86 ± 10.72%.

Notably, SANE-TZL exhibited superior therapeutic performance. The low-dose group (0.1 mg/kg) achieved a TGI of 41.81 ± 6.96%, matching the efficacy of free TZL at a significantly lower dose. The TGI for the middle (0.33 mg/kg) and high (1 mg/kg) dose SANE-TZL groups further increased to 50.33 ± 6.42% and 58.55 ± 5.13%, respectively (Table S3). Based on the individual tumor volume data (Table S4), the final average tumor volume of the high-dose group (0.546 ± 0.038 cm^3^) was approximately 2.6-fold smaller than that of the saline control group (1.414 ± 0.117 cm^3^) (Figure 6a,b,d). Individual comparisons of tumor volumes between SANE-TZL and saline or Free-TZL are further illustrated in Figure S5. Additionally, the blank carrier eSANE showed a TGI of 17.59%, suggesting a synergistic contribution from the stearic acid matrix.

Safety assessments indicated that all treatment groups maintained steady body weight gain throughout the study (Figure 6c). Histopathological (H&E) staining of major organs (Figure 6e) and serum biochemical markers (e.g., ALT, AST, and CK; Figure S6) exhibited no significant differences compared to the control group (*p* > 0.05), confirming the excellent systemic biosafety of SANE-TZL at therapeutic dosages.

## 4. Discussion

### 4.1. SANE-TZL as an Efficient Platform to Deliver TZL

In this study, long-chain fatty acids with different degrees of saturation were systematically compared as nanoemulsion cores for TZL delivery [28]. Among these, the SANE showed distinct physicochemical advantages for formulating TZL. In the long-term stability study, OANE-TZL and LANE-TZL exhibited pronounced increases in particle size, which can be attributed to the relatively higher aqueous solubility of unsaturated or shorter-chain fatty acids and the consequent enhancement of Ostwald ripening, i.e., the preferential migration of material from smaller to larger droplets [29,30]. By contrast, stearic acid, a long-chain saturated fatty acid, forms a highly hydrophobic and densely packed core that is less prone to molecular diffusion, thereby attenuating Ostwald ripening and conferring improved size stability over time. Together with the negative zeta potentials (approximately −15 to −30 mV) of all LCFANE-TZL formulations, which provide electrostatic repulsion against droplet coalescence, the compact stearic acid core appears to play a key role in maintaining colloidal stability during storage.

Regarding drug loading, SANE-TZL achieved an EE% of 71.42 ± 2.87% and a DL% of 6.43 ± 0.13%. It should be noted that during the 4-h dialysis used for EE determination, a degree of dynamic drug exchange between the lipid core and external medium may occur, potentially leading to a conservative estimation of the actual loading capacity. Nonetheless, the strictly standardized dialysis conditions across all formulations ensure the validity of our comparative analysis between different fatty acid-based platforms.

Despite these negative surface charges, which might theoretically pose an electrostatic barrier to cell membrane interaction, SANE-TZL demonstrated remarkably high cellular uptake. This efficient internalization can be explained by several coordinated mechanisms. First, in serum-containing biological media, the nanoparticles rapidly adsorb a “protein corona” that effectively shields the initial negative charge and provides biological ligands for cellular recognition [31]. Second, the high lipophilicity of the stearic acid core promotes hydrophobic partitioning and potential membrane fusion, allowing the nanocarriers to overcome weak electrostatic repulsion [32]. Most importantly, stearic acid acts as a functional ligand for fatty acid transporters such as CD36 (FAT), which are often overexpressed in cancer cells, thereby driving receptor-mediated active endocytosis regardless of the unfavorable surface potential [33].

The release profiles further support the suitability of SANE-TZL for tumor-targeted delivery. LCFANE-TZL exhibited sustained drug release at pH 6.5, a condition representative of the mildly acidic tumor microenvironment, suggesting that the formulation can gradually liberate TZL in situ and help maintain effective drug levels within the tumor region. Although appreciable release was also observed at pH 7.4, indicating that a fraction of TZL may be released during systemic circulation, several studies have shown that nanoformulations can still provide superior intratumoral accumulation and therapeutic benefit compared with conventional formulations, even in the presence of partial premature release [34,35]. This is largely due to their ability to circulate for extended periods, exploit the enhanced permeability and retention (EPR) effect of leaky tumor vasculature, and distribute within the expanded interstitial space of breast tumors [36,37]. In this context, TZL released into the bloodstream may help sustain systemic exposure and facilitate early diffusion into peritumoral regions, while the nanoemulsions that reach the tumor microenvironment can further release the remaining encapsulated drug under mildly acidic conditions. Such a two-phase delivery pattern—systemic exposure combined with pH-modulated intratumoral release—may collectively contribute to the improved antitumor activity and reduced systemic toxicity observed with SANE-TZL.

### 4.2. In Vitro Anticancer Effect and Induction of “Synthetic BRCAness”

A series of in vitro assays indicated that SANE-TZL substantially enhances the cytotoxicity of TZL against BRCA-proficient breast cancer cells. This enhancement appears to be driven, at least in part, by the altered cellular uptake behavior conferred by the nanoemulsion carrier. Despite its smaller particle size (~66 nm), LANE-TZL showed the weakest pro-apoptotic effect in MCF-7 cells, whereas SANE-TZL achieved the highest intracellular TZL accumulation, approximately 4.9-fold higher than that of the free drug. This finding suggests that the composition of the fatty acid core is more critical than size alone for cellular internalization [38]. The structural compatibility between long-chain saturated stearic acid and the phospholipid bilayer of the cell membrane may facilitate endocytosis-mediated uptake of SANE-TZL, thereby increasing intracellular drug exposure and contributing to the observed 8.4-fold reduction in IC_50_ compared with free TZL.

In addition to acting as a carrier, stearic acid itself may provide intrinsic biological support to the overall antiproliferative effect. Previous reports have shown that stearic acid can directly inhibit cancer cell growth by inducing endoplasmic reticulum (ER) stress, activating the unfolded protein response (UPR), and promoting apoptosis, as well as by modulating the recruitment of pro-tumorigenic immune cells in the tumor microenvironment [39,40,41,42]. These mechanisms are consistent with the 17.59% cell growth inhibition observed in the blank eSANE group in our study and may partially explain the enhanced apoptosis rate (up to 89.64%) in the SANE-TZL group. Furthermore, the reported ability of stearic acid to upregulate *CASP3* expression aligns with our observation of augmented apoptotic signaling in SANE-TZL-treated cells, supporting the concept of carrier-assisted chemosensitization.

Mechanistically, our RT-qPCR data suggest that SANE-TZL may exert its superior potency, at least in part, by modulating key components of the DDR pathway and thereby increasing functional vulnerability to PARP inhibition in BRCA-proficient cells. Free TZL, when administered to BRCA-proficient MCF-7 cells, has been associated with a compensatory upregulation of *BRCA1* and *RAD51*, which can partially restore homologous recombination and limit the extent of synthetic lethality. In contrast, SANE-TZL delivery led to a concurrent downregulation of *BRCA1*, *RAD51*, and the interstrand cross-link repair marker *FANCI*, together with an upregulation of *SLFN11*, a marker of heightened sensitivity to DNA damage [43,44].

Notably, our results showed that SANE-TZL significantly suppressed *PARP1* mRNA expression compared to the control and free TZL groups. While TZL primarily acts by trapping PARP proteins on DNA to create lethal lesions, the reduction in *PARP1* levels observed here may further compromise the overall DNA repair capacity of the cell [45], especially when combined with the simultaneous downregulation of HR-related genes like *BRCA1* and *RAD51* [46]. This dual hit—suppressing both the target *PARP1* and its compensatory repair pathways—likely creates a severe “repair-deficient” environment. Such coordinated changes are compatible with a more DDR-compromised, “BRCA-like” state often referred to as synthetic BRCAness [47], in which PARP inhibition is expected to induce more extensive and less repairable DNA damage, thereby amplifying apoptosis.

It should be noted, however, that these mechanistic insights are primarily based on mRNA expression profiles and phenotypic readouts from a limited number of cell lines. Specifically, while mRNA downregulation of PARP1 was observed, the subsequent impact on PARP trapping and PAR (poly-ADP-ribose) levels—the definitive hallmarks of PARP inhibitor action—remains to be fully elucidated. Protein-level validation (e.g., PARP1, cleaved PARP, BRCA1, RAD51, FANCI, SLFN11, and γH2AX), PARP1 trapping assays, expansion to additional breast cancer models, and gain-/loss-of-function studies will be required in future work to more definitively establish the causal links between SANE-TZL-induced gene expression changes and features of synthetic BRCAness.

### 4.3. In Vivo Antitumor Efficacy and Clinical Translational Safety

The in vivo data obtained in the MDA-MB-231 xenograft model further support the therapeutic promise of SANE-TZL. MDA-MB-231 is a highly invasive, BRCA-proficient, triple-negative breast cancer cell line that displays BRCAness-like molecular features and is widely used to model aggressive, treatment-refractory breast cancer in vivo. This makes it a relevant model to evaluate whether a functional nanoemulsion can enhance the efficacy of PARP inhibition beyond germline BRCA-mutant disease [47].

In this model, SANE-TZL produced robust TGI, with the high-dose group achieving a TGI of 58.55%, which exceeds commonly used thresholds for significant antitumor activity in nanomedicine studies. Importantly, SANE-TZL achieved comparable or superior tumor suppression to free TZL at substantially lower TZL-equivalent doses, indicating a clear dose-sparing effect. For example, SANE-TZL at 0.1 mg/kg yielded a TGI similar to that of free TZL at 0.33 mg/kg, and further dose escalation to 1.0 mg/kg led to even greater tumor inhibition. Such dose reduction is particularly relevant in the context of TZL-associated hematological toxicities, as it may allow effective tumor control at doses below those that typically limit clinical use [48].

The improved in vivo performance of SANE-TZL likely arises from the convergence of several formulation- and biology-driven advantages. First, the intermediate particle size (~118 nm) is suitable for exploiting the EPR effect, enabling preferential extravasation into tumor tissue through leaky vasculature and subsequent retention in the tumor interstitium. Second, the stearic acid-based core provides excellent biocompatibility and may contribute auxiliary antitumor effects, as reflected by the measurable TGI of the blank eSANE group. Third, the pH-responsive release behavior of LCFANE-TZL supports sustained drug liberation in the mildly acidic tumor microenvironment, potentially maintaining higher intratumoral TZL concentrations over time.

Safety is a critical determinant of translational feasibility for any PARP inhibitor–based regimen. Conventional TZL therapy is frequently complicated by hematological adverse events, including anemia and neutropenia, which constrain dose escalation and long-term administration. In our study, mice treated with SANE-TZL, including the 1.0 mg/kg group, maintained stable or slightly increased body weights throughout the treatment period. Histopathological analysis of major organs (liver, lung, heart, kidney, and spleen) revealed no apparent treatment-related damage, and serum biochemical parameters associated with hepatic and cardiac function (e.g., ALT, AST, and CK-MB) remained within normal physiological ranges. These findings suggest that SANE-TZL can redistribute TZL away from healthy critical organs while maintaining high drug exposure in tumors, thus improving the therapeutic index.

Collectively, the in vivo results demonstrate that SANE-TZL can suppress tumor growth effectively in a clinically relevant, aggressive TNBC model under BRCA-proficient conditions, while maintaining a favorable safety profile and offering the potential for dose reduction relative to free TZL. This provides encouraging preclinical evidence that functional stearic acid-based nanoemulsions may help extend the clinical benefits of PARP inhibition to a broader population of patients with BRCAness-like breast cancers.

## 5. Conclusions

In summary, we developed a stearic acid-based nanoemulsion (SANE-TZL) to improve TZL delivery and address limitations related to its poor aqueous solubility and systemic toxicity. The SANE carrier provided good colloidal stability, pH-responsive release, and enhanced cellular uptake of TZL. In breast cancer models, SANE-TZL markedly increased the antiproliferative and pro-apoptotic activity of TZL versus the free drug, with reduced IC_50_ values in vitro and dose-dependent tumor growth inhibition in vivo at lower TZL-equivalent doses, while maintaining a favorable safety profile. Molecular analyses indicated that SANE-TZL modulates DDR-related genes in a pattern consistent with increased susceptibility to PARP inhibition. Overall, these findings suggest that functional stearic acid-based nanoemulsions can enhance the antitumor efficacy and therapeutic index of TZL and warrant further mechanistic and translational investigation.

## Figures and Tables

**Table 1:** Detailed compositions of LCFANE-TZL.

Empty LCFANE		LCFANE-TZL	
Components	Weight Ratio (%)	Components	Weight Ratio (%)
DSPC	55	DSPC	50
Tween-80	20	Tween-80	18
OA/LA/SA	25	OA/LA/SA	23
TZL	0	TZL	9

## Data Availability

The original contributions presented in this study are included in the article/Supplementary Material. Further inquiries can be directed to the corresponding author(s).

## Supplementary Materials

The following supporting information can be downloaded at https://www.mdpi.com/article/10.3390/pharmaceutics18030378/s1. Figure S1: UV standard curve and encapsulation efficiency (*EE*%) optimization data of LCFANE-TZL; Figure S2: Qualitative and quantitative evaluation of cellular uptake in MCF-7 cells; Figure S3: Cytotoxicity of different LCFANE-TZL formulations in MDA-MB-231 cells; Figure S4: RT-qPCR analysis of DNA damage response-related gene expression in MCF-7 cells; Figure S5: RT-qPCR analysis of DNA damage response-related gene expression in MDA-MB-231 cells; Figure S6: Individual and combined tumor growth curves across treatment groups; Figure S7: Serum biochemical analysis for biosafety evaluation; Table S1: Primer sequences used for RT-qPCR analysis; Table S2: Statistical analysis of total apoptotic rates in MCF-7 and MDA-MB-231 cells; Table S3: Tumor growth inhibition (*TGI*) rates across different treatment groups; Table S4: Individual tumor volume data for each mouse in all treatment groups.

## Abbreviations

The following abbreviations are used in this manuscript:

ER	Estrogen receptor
PR	Progesterone receptor
HER2	Human epidermal growth factor receptor 2
BRCA	Breast cancer susceptibility gene
BRCA1/2	Breast cancer susceptibility gene 1/2
TNBC	Triple-negative breast cancer
PARP	Poly(ADP-ribose) polymerase
BCS	Biopharmaceutics Classification System
DDR	DNA damage response
ER (cellular)	Endoplasmic reticulum
UPR	Unfolded protein response
EPR	Enhanced permeability and retention
NE/NEs	Nanoemulsion/nanoemulsions
LCFA	Long-chain fatty acid
LCFANEs	Long-chain fatty acid nanoemulsions
SA	Stearic acid
TZL	Talazoparib
SANE	Stearic acid-based nanoemulsion
SANE-TZL	Stearic acid-based nanoemulsion-loaded talazoparib
OA	Oleic acid
LA	Linoleic acid
DSPC	Distearoyl phosphatidylcholine
DMSO	Dimethyl sulfoxide
DMEM	Dulbecco’s Modified Eagle Medium
PBS	Phosphate-buffered saline
FBS	Fetal bovine serum
CCK-8	Cell Counting Kit-8
DEPC	Diethyl pyrocarbonate
HCl	Hydrochloric acid
CHCl_3_	Chloroform
IPA	Isopropanol
TEM	Transmission electron microscopy
DLS	Dynamic light scattering
PDI	Polydispersity index
EE	Encapsulation efficiency
TGI	Tumor growth inhibition
RFL	Relative fluorescence level
OD	Optical density
RT-qPCR	Reverse transcription–quantitative polymerase chain reaction
cDNA	Complementary DNA
H&E	Hematoxylin and eosin
CASP3	Caspase-3
CCND1	Cyclin D1
CDH1	Cadherin 1 (E-cadherin)
FANCI	Fanconi anemia complementation group I
GAPDH	Glyceraldehyde-3-phosphate dehydrogenase
ALT	Alanine aminotransferase
AST	Aspartate aminotransferase
CK-MB	Creatine kinase-MB
SD	Standard deviation
ANOVA	Analysis of variance
HSD	Honestly Significant Difference

## References

[1] Johnson K.S., Conant E.F., Soo M.S. (2021). Molecular Subtypes of Breast Cancer: A Review for Breast Radiologists. J. Breast Imaging.

[2] Miki Y., Swensen J., Shattuck-Eidens D., Futreal P.A., Harshman K., Tavtigian S., Liu Q., Cochran C., Bennett L.M., Ding W. (1994). A Strong Candidate for the Breast and Ovarian Cancer Susceptibility Gene *BRCA1*. Science.

[3] Patel K.J., Lee H., Corcoran A., Thistlethwaite F.C., Evans M.J., Colledge W.H., Friedman L.S., Ponder B.A.J., Venkitaraman A.R. (1998). Involvement of Brca2 in DNA Repair. Mol. Cell.

[4] Moynahan M.E., Chiu J.W., Koller B.H., Jasin M. (1999). Brca1 Controls Homology-Directed DNA Repair. Mol. Cell.

[5] Moynahan M.E., Pierce A.J., Jasin M. (2001). BRCA2 Is Required for Homology-Directed Repair of Chromosomal Breaks. Mol. Cell.

[6] Xiong N., Wu H., Yu Z. (2024). Advancements and Challenges in Triple-Negative Breast Cancer: A Comprehensive Review of Therapeutic and Diagnostic Strategies. Front. Oncol..

[7] Farmer H., McCabe N., Lord C.J., Tutt A.N.J., Johnson D.A., Richardson T.B., Santarosa M., Dillon K.J., Hickson I., Knights C. (2005). Targeting the DNA Repair Defect in BRCA Mutant Cells as a Therapeutic Strategy. Nature.

[8] Evans K.W., Yuca E., Akcakanat A., Scott S.M., Arango N.P., Zheng X., Chen K., Tapia C., Tarco E., Eterovic A.K. (2017). A Population of Heterogeneous Breast Cancer Patient-Derived Xenografts Demonstrate Broad Activity of PARP Inhibitor in BRCA1/2 Wild-Type Tumors. Clin. Cancer Res..

[9] Murai J., Huang S.-Y.N., Renaud A., Zhang Y., Ji J., Takeda S., Morris J., Teicher B., Doroshow J.H., Pommier Y. (2014). Stereospecific PARP Trapping by BMN 673 and Comparison with Olaparib and Rucaparib. Mol. Cancer Ther..

[10] Livraghi L., Garber J.E. (2015). PARP Inhibitors in the Management of Breast Cancer: Current Data and Future Prospects. BMC Med..

[11] Lee K.-H., Sohn J., Goodwin A., Usari T., Lanzalone S., Im S.-A., Kim S.-B. (2021). Talazoparib Versus Chemotherapy in Patients with HER2-Negative Advanced Breast Cancer and a Germline BRCA1/2 Mutation Enrolled in Asian Countries: Exploratory Subgroup Analysis of the Phase III EMBRACA Trial. Cancer Res. Treat..

[12] De Bono J., Ramanathan R.K., Mina L., Chugh R., Glaspy J., Rafii S., Kaye S., Sachdev J., Heymach J., Smith D.C. (2017). Phase I, Dose-Escalation, Two-Part Trial of the PARP Inhibitor Talazoparib in Patients with Advanced Germline *BRCA1/2* Mutations and Selected Sporadic Cancers. Cancer Discov..

[13] Wang B., Chu D., Feng Y., Shen Y., Aoyagi-Scharber M., Post L.E. (2016). Discovery and Characterization of (8*S*,9*R*)-5-Fluoro-8-(4-Fluorophenyl)-9-(1-Methyl-1*H*-1,2,4-Triazol-5-Yl)-2,7,8,9-Tetrahydro-3*H*-Pyrido[4,3,2-de]Phthalazin-3-One (BMN 673, Talazoparib), a Novel, Highly Potent, and Orally Efficacious Poly(ADP-Ribose) Polymerase-1/2 Inhibitor, as an Anticancer Agent. J. Med. Chem..

[14] Zhang D., Baldwin P., Leal A.S., Carapellucci S., Sridhar S., Liby K.T. (2019). A Nano-Liposome Formulation of the PARP Inhibitor Talazoparib Enhances Treatment Efficacy and Modulates Immune Cell Populations in Mammary Tumors of BRCA-Deficient Mice. Theranostics.

[15] Baldwin P., Likhotvorik R., Baig N., Cropper J., Carlson R., Kurmasheva R., Sridhar S. (2019). Nanoformulation of Talazoparib Increases Maximum Tolerated Doses in Combination with Temozolomide for Treatment of Ewing Sarcoma. Front. Oncol..

[16] Yu X., Ren X., Liang X., Tang Y. (2018). Roles of Fatty Acid Metabolism in Tumourigenesis: Beyond Providing Nutrition (Review). Mol. Med. Rep..

[17] Röhrig F., Schulze A. (2016). The Multifaceted Roles of Fatty Acid Synthesis in Cancer. Nat. Rev. Cancer.

[18] Camarda R., Zhou A.Y., Kohnz R.A., Balakrishnan S., Mahieu C., Anderton B., Eyob H., Kajimura S., Tward A., Krings G. (2016). Inhibition of Fatty Acid Oxidation as a Therapy for MYC-Overexpressing Triple-Negative Breast Cancer. Nat. Med..

[19] Woo J.O., Misran M., Lee P.F., Tan L.P. (2014). Development of a Controlled Release of Salicylic Acid Loaded Stearic Acid-Oleic Acid Nanoparticles in Cream for Topical Delivery. Sci. World J..

[20] Evans L.M., Cowey S.L., Siegal G.P., Hardy R.W. (2009). Stearate Preferentially Induces Apoptosis in Human Breast Cancer Cells. Nutr. Cancer.

[21] Roy A., Nishchaya K., Rai V.K. (2022). Nanoemulsion-Based Dosage Forms for the Transdermal Drug Delivery Applications: A Review of Recent Advances. Expert Opin. Drug Deliv..

[22] Pretor S., Bartels J., Lorenz T., Dahl K., Finke J.H., Peterat G., Krull R., Al-Halhouli A.T., Dietzel A., Büttgenbach S. (2015). Cellular Uptake of Coumarin-6 under Microfluidic Conditions into HCE-T Cells from Nanoscale Formulations. Mol. Pharm..

[23] Pai Bellare G., Sankar Patro B. (2022). Resveratrol Sensitizes Breast Cancer to PARP Inhibitor, Talazoparib through Dual Inhibition of AKT and Autophagy Flux. Biochem. Pharmacol..

[24] Pai Bellare G., Saha B., Patro B.S. (2021). Targeting Autophagy Reverses de Novo Resistance in Homologous Recombination Repair Proficient Breast Cancers to PARP Inhibition. Br. J. Cancer.

[25] Baldwin P., Ohman A.W., Medina J.E., McCarthy E.T., Dinulescu D.M., Sridhar S. (2019). Nanoformulation of Talazoparib Delays Tumor Progression and Ascites Formation in a Late Stage Cancer Model. Front. Oncol..

[26] Baldwin P., Yang S., Orriols A., Wang S., Brown N., Sridhar S. (2024). A Nano-Cocktail of the PARP Inhibitor Talazoparib and CDK Inhibitor Dinaciclib for the Treatment of Triple Negative Breast Cancer. Cancer Nanotechnol..

[27] Kizilbash S.H., Gupta S.K., Chang K., Kawashima R., Parrish K.E., Carlson B.L., Bakken K.K., Mladek A.C., Schroeder M.A., Decker P.A. (2017). Restricted Delivery of Talazoparib Across the Blood–Brain Barrier Limits the Sensitizing Effects of PARP Inhibition on Temozolomide Therapy in Glioblastoma. Mol. Cancer Ther..

[28] Mou D., Chen H., Du D., Mao C., Wan J., Xu H., Yang X. (2008). Hydrogel-Thickened Nanoemulsion System for Topical Delivery of Lipophilic Drugs. Int. J. Pharm..

[29] Kabalnov A. (2001). Ostwald Ripening and Related Phenomena. J. Dispers. Sci. Technol..

[30] Solans C., Izquierdo P., Nolla J., Azemar N., Garcia-Celma M.J. (2005). Nano-Emulsions. Curr. Opin. Colloid Interface Sci..

[31] Monopoli M.P., Åberg C., Salvati A., Dawson K.A. (2012). Biomolecular Coronas Provide the Biological Identity of Nanosized Materials. Nat. Nanotechnol..

[32] Verma A., Stellacci F. (2010). Effect of Surface Properties on Nanoparticle–Cell Interactions. Small.

[33] Glatz J.F.C., Luiken J.J.F.P. (2018). Dynamic Role of the Transmembrane Glycoprotein CD36 (SR-B2) in Cellular Fatty Acid Uptake and Utilization. J. Lipid Res..

[34] Danhier F. (2016). To Exploit the Tumor Microenvironment: Since the EPR Effect Fails in the Clinic, What Is the Future of Nanomedicine?. J. Control. Release.

[35] Bertrand N., Wu J., Xu X., Kamaly N., Farokhzad O.C. (2014). Cancer Nanotechnology: The Impact of Passive and Active Targeting in the Era of Modern Cancer Biology. Adv. Drug Deliv. Rev..

[36] De Visser K.E., Joyce J.A. (2023). The Evolving Tumor Microenvironment: From Cancer Initiation to Metastatic Outgrowth. Cancer Cell.

[37] Maeda H. (2001). The Enhanced Permeability and Retention (EPR) Effect in Tumor Vasculature: The Key Role of Tumor-Selective Macromolecular Drug Targeting. Adv. Enzym. Regul..

[38] Gupta A., Eral H.B., Hatton T.A., Doyle P.S. (2016). Nanoemulsions: Formation, Properties and Applications. Soft Matter.

[39] Ogura J., Yamanoi K., Ishida K., Nakamura E., Ito S., Aoyama N., Nakanishi Y., Menju T., Kawaguchi K., Hosoe Y. (2024). A Stearate-Rich Diet and Oleate Restriction Directly Inhibit Tumor Growth via the Unfolded Protein Response. Exp. Mol. Med..

[40] Tsenkova M., Brauer M., Pozdeev V.I., Kasakin M., Busi S.B., Schmoetten M., Cheung D., Meyers M., Rodriguez F., Gaigneaux A. (2025). Ketogenic Diet Suppresses Colorectal Cancer through the Gut Microbiome Long Chain Fatty Acid Stearate. Nat. Commun..

[41] Hardy S., El-Assaad W., Przybytkowski E., Joly E., Prentki M., Langelier Y. (2003). Saturated Fatty Acid-Induced Apoptosis in MDA-MB-231 Breast Cancer Cells. J. Biol. Chem..

[42] El-Assaad W., Buteau J., Peyot M.-L., Nolan C., Roduit R., Hardy S., Joly E., Dbaibo G., Rosenberg L., Prentki M. (2003). Saturated Fatty Acids Synergize with Elevated Glucose to Cause Pancreatic β-Cell Death. Endocrinology.

[43] Murai J., Tang S.-W., Leo E., Baechler S.A., Redon C.E., Zhang H., Al Abo M., Rajapakse V.N., Nakamura E., Jenkins L.M.M. (2018). SLFN11 Blocks Stressed Replication Forks Independently of ATR. Mol. Cell.

[44] Okamoto Y., Abe M., Mu A., Tempaku Y., Rogers C.B., Mochizuki A.L., Katsuki Y., Kanemaki M.T., Takaori-Kondo A., Sobeck A. (2021). *SLFN11* Promotes Stalled Fork Degradation That Underlies the Phenotype in Fanconi Anemia Cells. Blood.

[45] Ray Chaudhuri A., Nussenzweig A. (2017). The Multifaceted Roles of PARP1 in DNA Repair and Chromatin Remodelling. Nat. Rev. Mol. Cell Biol..

[46] Scanlon S.E., Hegan D.C., Sulkowski P.L., Glazer P.M. (2018). Suppression of Homology-Dependent DNA Double-Strand Break Repair Induces PARP Inhibitor Sensitivity in *VHL*-Deficient Human Renal Cell Carcinoma. Oncotarget.

[47] Lord C.J., Ashworth A. (2016). BRCAness Revisited. Nat. Rev. Cancer.

[48] Litton J.K., Rugo H.S., Ettl J., Hurvitz S.A., Gonçalves A., Lee K.-H., Fehrenbacher L., Yerushalmi R., Mina L.A., Martin M. (2018). Talazoparib in Patients with Advanced Breast Cancer and a Germline *BRCA* Mutation. N. Engl. J. Med..

