# Supplementary material for: Development of Stearic Acid Nanoemulsion for Therapeutic Delivery of Talazoparib Against Breast Cancer
Source: Pharmaceutics. 2026 Mar 19;18(3):378. doi: 10.3390/pharmaceutics18030378 (PMC13029586; doi:10.3390/pharmaceutics18030378)
Supplement: Supplementary file 1 [file pharmaceutics-18-00378-s001.zip › pharmaceutics-4189203-supplementary.pdf]

Supplementary Materials

Development of Stearic Acid Nanoemulsion for Therapeutic Delivery of Talazoparib against Breast Cancer

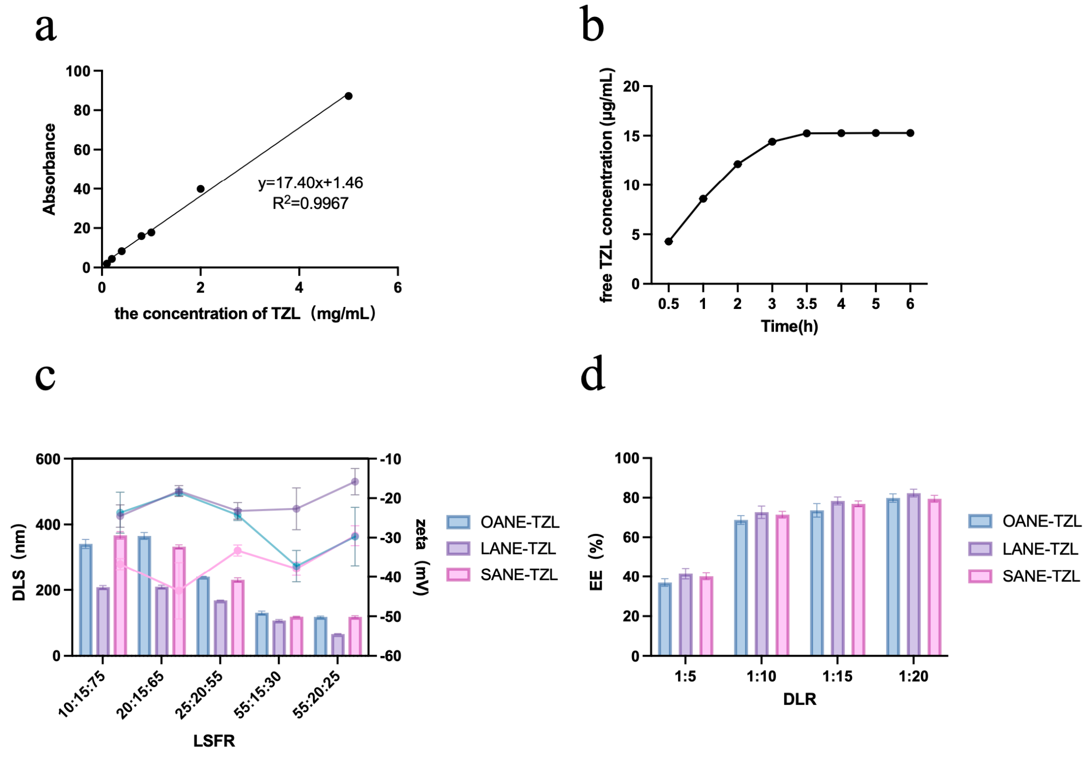

Figure S1. UV standard curve and encapsulation efficiency (EE%) of LCFANE-TZL, as well as formulation optimization data.(a) UV absorbance standard curve of TZL in PBS at λ = 311 nm(R^2 = 0.9967).(b) Dialysis equilibrium verification for EE% determination with SANE-TZL as the representative: the concentration of free TZL in external PBS medium (pH 7.4, 37 °C, 100 rpm) at different time points.(c) Particle size (left Y-axis, nm) and zeta potential (right Y-axis, mV) of LCFANE-TZL formulations with different lipid-surfactant-fatty acid mass ratios (LSFR: 10:15:75, 20:15:65, 25:20:55, 55:15:30, 55:20:25) at a fixed drug-lipid ratio (DLR) of 1:10.(d) EE% of LCFANE-TZL formulations with different DLR (1:5, 1:10, 1:15, 1:20) at the optimal LSFR of 55:20:25. Data are presented as mean ± SD (n = 3).

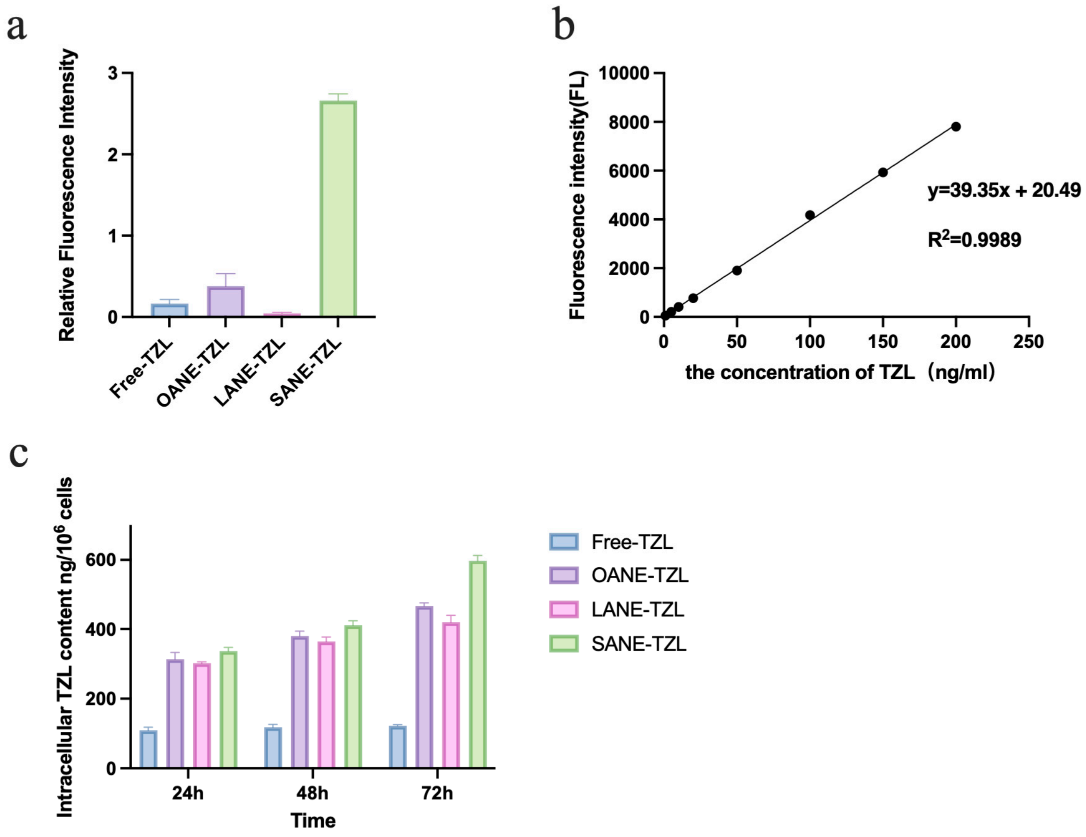

Figure S2. Qualitative and quantitative evaluation of cellular uptake in MCF-7 cells. (a) Preliminary qualitative assessment of carrier internalization using DiO-labeled Free-TZL and LCFANE-TZL after 48 h of incubation. (b) Calibration curve of TZL fluorescence intensity in blank cell lysate. (b) Time-dependent quantitative analysis of intracellular drug accumulation (24, 48, and 72 h) measured via the intracellular TZL content. Data are presented as mean ± SD (n = 3).

a

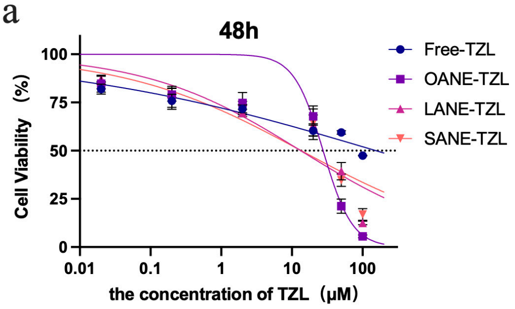

b

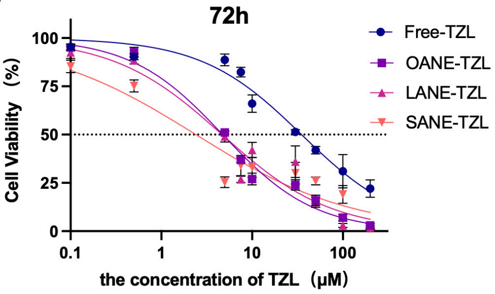

c

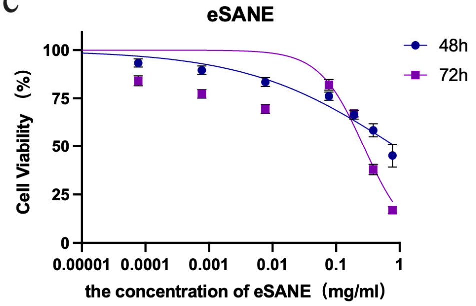

d

Time / Group	IC_{50} (μM)	
	48h	72h
Free-TZL	154.40	35.03
OANE-TZL	27.63	4.73
LANE-TZL	13.32	4.67
SANE-TZL	13.57	2.40

Figure S3. Cytotoxicity of Free-TZL and LCFANE-TZL in MDA-MB-231 cells. (a–b) Cell viability of MDA-MB-231 cells after treatment with Free-TZL and various LCFANE-TZL formulations for (a) 48 h and (b) 72 h, determined by CCK-8 assay.(c) Cell viability after treatment with eSANE for 48 h and 72 h. (d) IC_{50} values of Free-TZL and LCFANE-TZL at 48 h and 72 h.Data are pre sented as mean ± SD (n = 3).

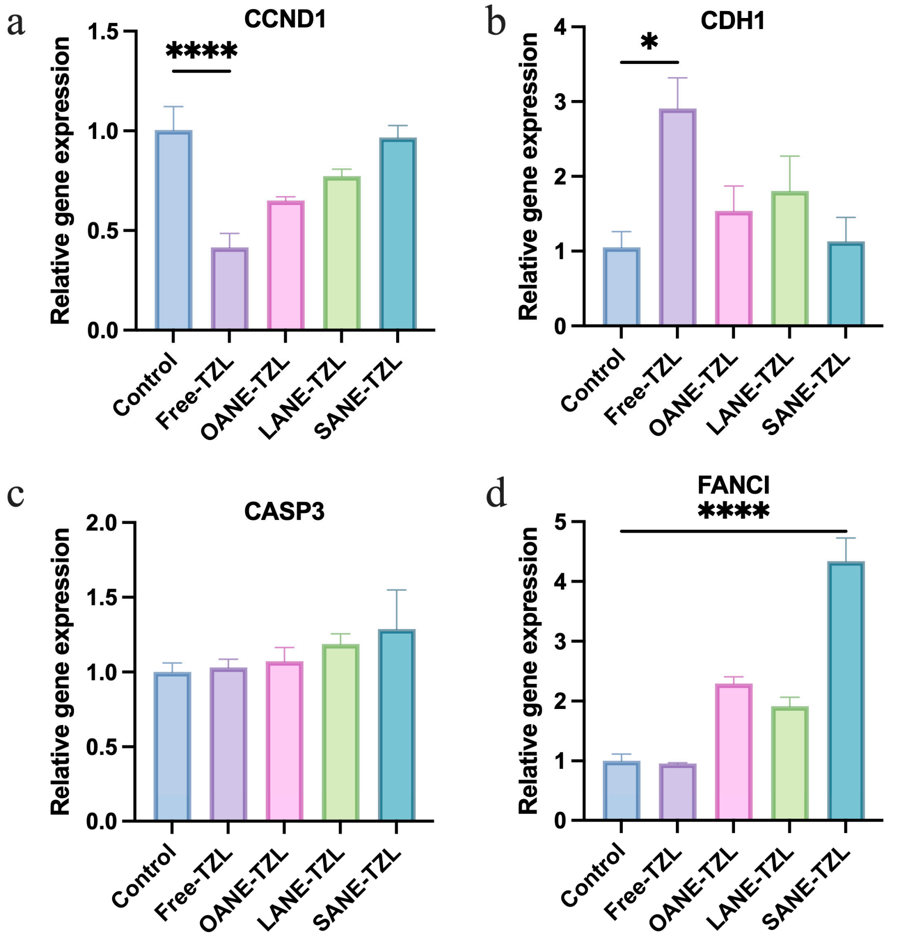

Figure S4. Supplemental RT-qPCR analysis of gene expression in MCF-7 cells. (a–d) Relative mRNA expression levels of (a) *Cyclin D1*, (b) *E-cadherin*, (c) *CASP3*, and (d) *FANCI* in MCF-7 cells treated with Free-TZL and various LCFANE-TZL formulations.Data are presented as mean ± SD (n = 3).Significant differences are indicated as * p<0.05, ** p<0.01, ****p＜0.0001.

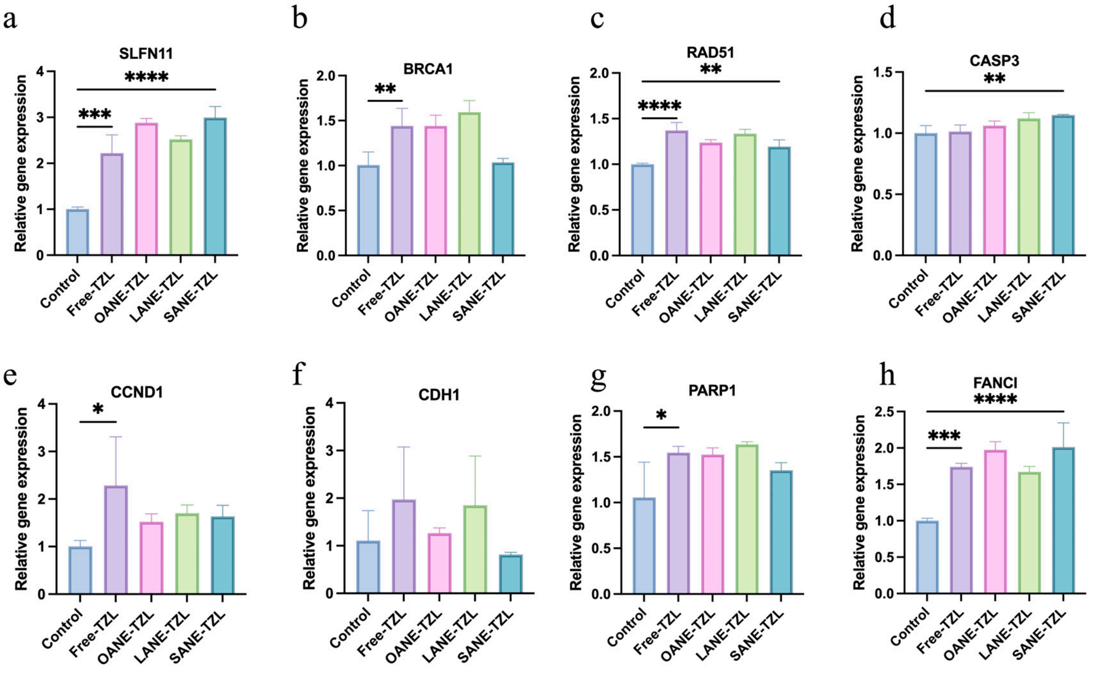

Figure S5. RT-qPCR analysis of gene expression in MDA-MB-231 cells. (a–d) Relative mRNA expression levels of (a) *SLFN11*, (b) *BRCA1*, (c) *RAD51*, (d) *CASP3,*(e) *CCND1*, (f) *CDH1*, (g) *PARP1*, and (h) *FANCI* in MDA-MB-231 cells treated with Free-TZL and various LCFANE-TZL formulations.Data are presented as mean ± SD (n = 3).Significant differences are indicated as * $p<0.05$, ** $p<0.01$, ***$p<0.001$, ****$p<0.0001$.

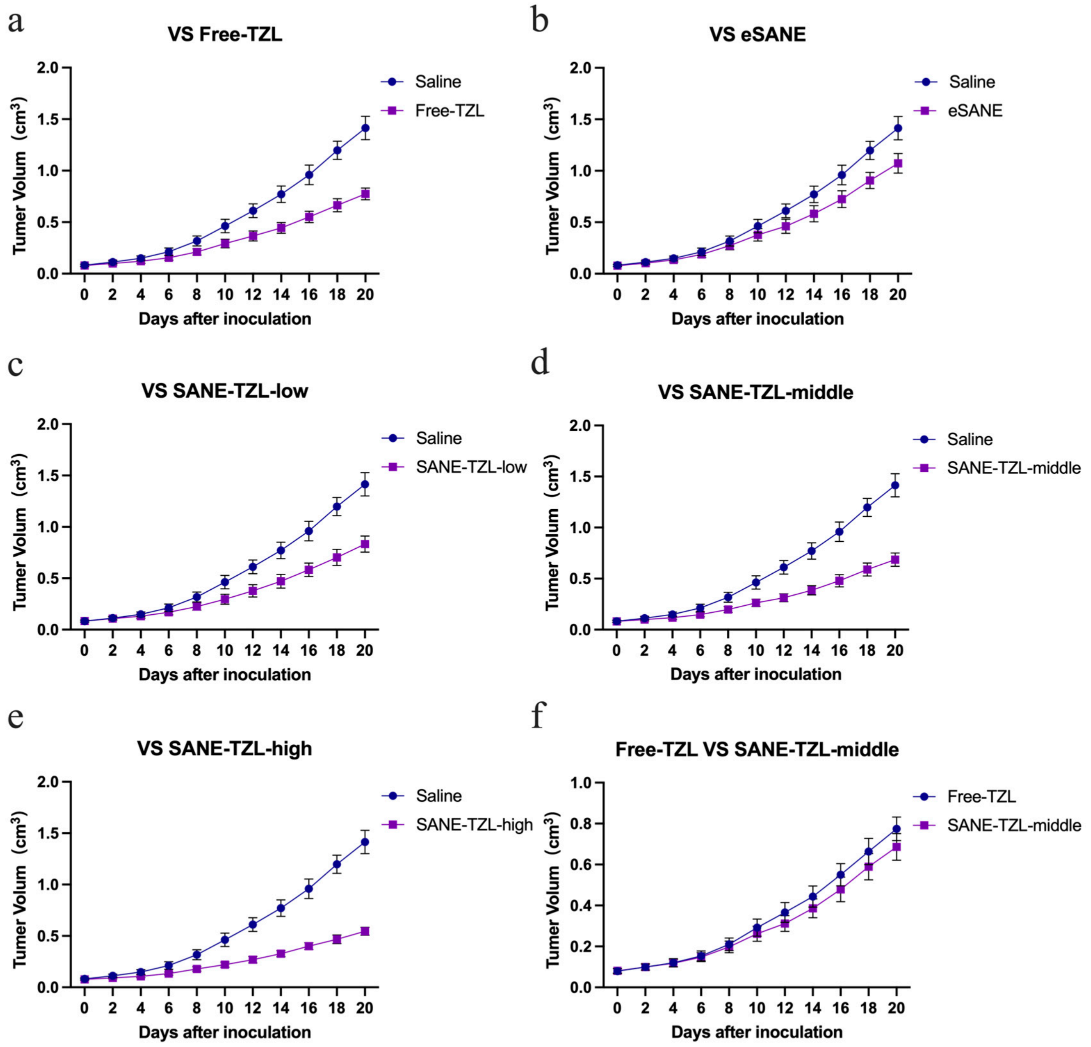

Figure S6. Individual and combined tumor growth curves. (a–e) Pairwise comparison of tumor volume kinetics between Saline and: (a) Free-TZL, (b) eSANE, (c) SANE-TZL-low, (d) SANE-TZL-middle, and (e) SANE-TZL-high. (f) Combined growth curves of Saline, Free-TZL, and middle/high-dose SANE-TZL groups. Data are presented as mean ± SD (n = 6).

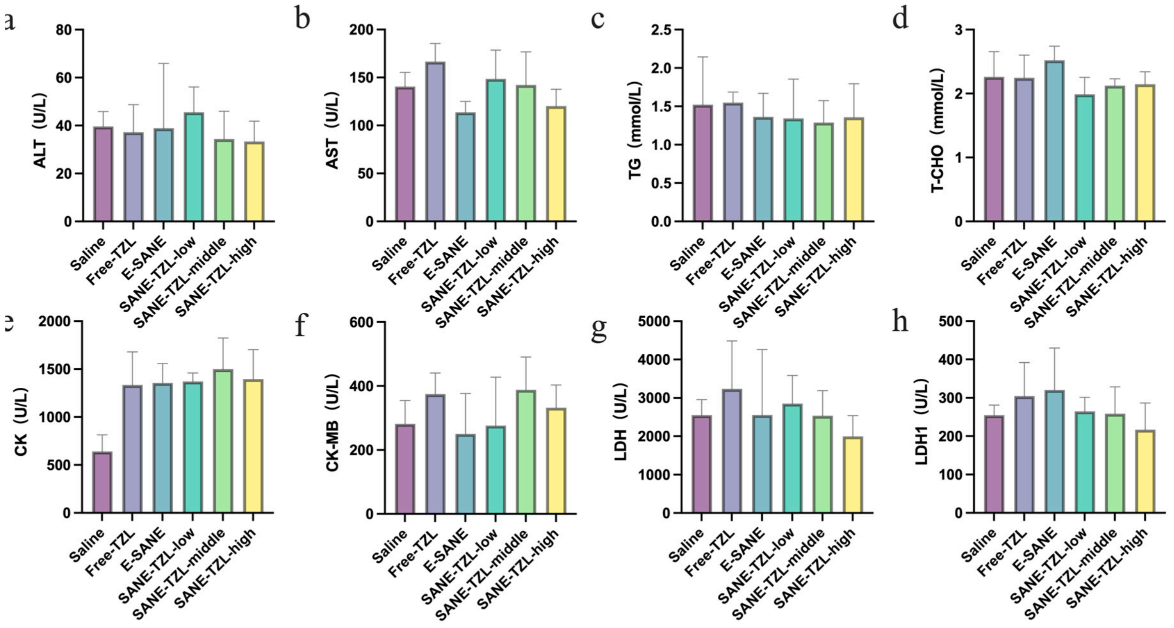

Figure S7. Serum biochemical analysis for biosafety evaluation. Levels of liver function markers (a) ALT and (b) AST, lipid metabolic markers (c) TG and (d) T-CHO, and myocardial enzyme markers (e) CK, (f) CK-MB, (g) LDH, and (h) LDH1 in mice following different treatments. No significant systemic toxicity was observed across all LCFANE-TZL groups compared to the Saline control. Data are presented as mean ± SD (n = 6).

Table S1 Details of the gene sequences used

Gene	Sequences(5' → 3')
GAPDH	F：GACCCCTTCATTGACCTCAAC<br>R：CTTCTGAGTGGCAGTGATGG
RAD51	F：GTTGCCTATGCGCCAAAGAA<br>R：GATCTCTGACCGCCTTTGGT
FANCI	F：TGCTTTCAGCCACTTCTCCA<br>R：TGCTGAAGTTCTGTGGTGGA
CDH1	F：TGCCCAGAAAATGAAAAAGG<br>R：GTGTATGTGGCAATGCGTTC
BRCA1	F：GAAACCGTGCCAAAAGACTTC<br>R：CCAAGGTTAGAGAGTTGGACAC
CCND1	F：GCTGCGAAGTGGAAACCATC<br>R：CCTCCTTCTGCACACATTTGAA
PARP1	F：GCTTCAGCCTCCTTGCTACA<br>R：TTCGCCACTTCATCCACTCC
CASP3	F：AGAACTGGACTGTGGCATTG<br>R：ATCTGTTGCCACCTTTCGGT
SLFN11	F：GCAACCCCACCATCTGCTTA<br>R：GACTAGATCCTGGGGCATGG

Table S2. Statistical correlation and apoptotic enhancement rates of LCFANE-TZL formulations.

Group		P value	increase (%)
Control VS	Free-TZL	0.0002	62.68
	OANE-TZL	<0.0001	195.36
	LANE-TZL	<0.0001	135.20
	SANE-TZL	<0.0001	206.04
Free-TZL VS	OANE-TZL	<0.0001	81.55
	LANE-TZL	<0.0001	44.58
	SANE-TZL	<0.0001	88.12

Note: "Increase (%)" indicates the percentage growth in the total apoptotic rate of the treatment group relative to the specified reference group (Control or Free-TZL).

Table S3. Tumor growth inhibition (TGI) rates across different treatment groups.

Group		TGI（%）（Mean ± SD）
Saline VS	Free-TZL	41.86 ± 10.72
	eSANE	17.59 ± 16.23
	SANE-TZL-low	41.81 ± 6.96
	SANE-TZL-middle	50.33 ± 6.42
	SANE-TZL-high	58.55 ± 5.13

Note: The Tumor Growth Inhibition (TGI) rate was calculated based on the tumor volume of the Saline control group at the end of the study. SANE-TZL-low, middle, and high groups correspond to dosages of 0.1, 0.33, and 1 mg/kg, respectively.Data are presented for six individual mice (n = 6) and summarized as Mean ± SD.

Table S4. Individual tumor volume data (cm^3) of mice in each group at the end of the treatment. Data are presented for six individual mice (n = 6) and summarized as Mean ± SD.

Group	Mouse 1	Mouse 2	Mouse 3	Mouse 4	Mouse 5	Mouse 6	Mean ± SD
Saline	1.400	1.512	1.316	1.589	1.371	1.298	1.414 ± 0.117
Free-TZL	0.753	0.883	0.779	0.734	0.724	0.772	0.774 ± 0.057
eSANE	1.125	0.956	0.996	1.161	1.017	1.180	1.073 ± 0.096
SANE-TZL-low	0.864	0.739	0.922	0.748	0.810	0.906	0.832 ± 0.080
SANE-TZL-middle	0.650	0.617	0.758	0.769	0.694	0.628	0.686 ± 0.067
SANE-TZL-high	0.592	0.503	0.547	0.511	0.589	0.533	0.546 ± 0.038
